# Systematic review of studies examining contribution of oral health variables to risk prediction models for undiagnosed Type 2 diabetes and prediabetes

**DOI:** 10.1002/cre2.515

**Published:** 2021-11-30

**Authors:** Ingrid Glurich, Neel Shimpi, Barb Bartkowiak, Richard L. Berg, Amit Acharya

**Affiliations:** ^1^ Center for Oral and Systemic Health Marshfield Clinic Research Institute Marshfield Wisconsin USA; ^2^ Marshfield Clinic GE Magnin Medical Library Marshfield Clinic Health System Marshfield Wisconsin USA; ^3^ Office of Research Computing and Analytics Marshfield Clinic Research Institute Marshfield Wisconsin USA; ^4^ Advocate Aurora Research Institute Advocate Aurora Health Downers Grove Illinois USA

**Keywords:** diabetes mellitus, oral health, risk assessment, systematic review

## Abstract

**Objective:**

To conduct systematic review applying “preferred reporting items for systematic reviews and meta‐analyses statement” and “prediction model risk of assessment bias tool” to studies examining the performance of predictive models incorporating oral health‐related variables as candidate predictors for projecting undiagnosed diabetes mellitus (Type 2)/prediabetes risk.

**Materials and Methods:**

Literature searches undertaken in PubMed, Web of Science, and Gray literature identified eligible studies published between January 1, 1980 and July 31, 2018. Systematically reviewed studies met inclusion criteria if studies applied multivariable regression modeling or informatics approaches to risk prediction for undiagnosed diabetes/prediabetes, and included dental/oral health‐related variables modeled either independently, or in combination with other risk variables.

**Results:**

Eligibility for systematic review was determined for seven of the 71 studies screened. Nineteen dental/oral health‐related variables were examined across studies. “Periodontal pocket depth” and/or “missing teeth” were oral health variables consistently retained as predictive variables in models across all systematically reviewed studies. Strong performance metrics were reported for derived models by all systematically reviewed studies. The predictive power of independently modeled oral health variables was marginally amplified when modeled with point‐of‐care biological glycemic measures in dental settings. Meta‐analysis was precluded due to high inter‐study variability in study design and population diversity.

**Conclusions:**

Predictive modeling consistently supported “periodontal measures” and “missing teeth” as candidate variables for predicting undiagnosed diabetes/prediabetes. Validation of predictive risk modeling for undiagnosed diabetes/prediabetes across diverse populations will test the feasibility of translating such models into clinical practice settings as noninvasive screening tools for identifying at‐risk individuals following demonstration of model validity within the defined population.

## 1 INTRODUCTION

More than 30 million adults (over 9% of the US population) have diabetes, and nearly one in three (approximately 84 million individuals) are prediabetic. The 2020 national statistics report on diabetic disease burden in the United States estimated that 10.5% of the population has diabetes with 21.4% of individuals with diabetes unaware of their status, while 34.5% of the population has prediabetes (Centers for Disease Control and Prevention, 2020). Like diabetes, periodontitis has also achieved epidemic status in the United States based on recently updated estimates of periodontitis prevalence projected by Eke et al. (2000) following examination of the National Health and Nutrition Examination Survey (NHANES) population data. Applying three case definitions, the authors defined a prevalence rate of 42%, a sixfold increase over diabetes prevalence. Among those with periodontitis, 7.8% met definitions aligning with severe periodontitis (Eke et al., 2000). A growing evidence base reinforced by both systematic review (Borgnakke et al., 2013) and meta‐analysis (Corbella et al., 2013; Wang et al., 2014) provides evidence of an adverse impact of periodontitis on glycemic control. The escalating prevalence of undiagnosed Type 2 diabetes mellitus and prediabetes (T2DM/prediabetes) and periodontitis in the global population are noteworthy in light of these biological interactions.

Bidirectional interaction and exacerbation between T2DM and periodontitis has been proposed (Casanova et al., 2014; Llambés et al., 2015; Sgolastra et al., 2013). Interaction between periodontitis and T2DM is supported by multiple reports of heightened periodontitis severity in association with uncontrolled T2DM and improved glycemic control following periodontitis treatment (Borgnakke et al., 2013; Casanova et al., 2014; Corbella et al., 2013; Demmer et al., 2010; Eke et al., 2000; Glurich et al., 2013; Llambés et al., 2015; Sgolastra et al., 2013; Wang et al., 2014). A study that conducted longitudinal monitoring of normoglycemic individuals with and without periodontitis noted an incremental fivefold increase in HbA1c levels overtime only in individuals with periodontitis (Demmer et al., 2010). This observation suggests a potential role for periodontitis in the pathogenesis of T2DM.

Underlying inflammatory processes are posited to contribute to the mutual exacerbation of T2DM and periodontitis (Glurich et al., 2013). Clinical challenges are faced by both medical and dental providers in managing patients with periodontitis and T2DM/prediabetes. Chronic local and systemic inflammation impedes efforts by medical providers to control hyperglycemia in diabetic patients with comorbid periodontitis, while dentists face challenges in preventing or resolving periodontitis in patients with uncontrolled blood sugar levels.

Importantly, both periodontitis and T2DM represent potentially modifiable conditions if detected and managed prior to becoming chronically established. Ideal clinical management strategies include an integrated medical‐dental care delivery approach that targets patient education and activation, lifestyle and behavioral interventions, early clinical intervention, and longitudinal monitoring for disease recurrence or progression (Glurich et al., 2017; Glurich, Schwei, et al., 2018; Shimpi et al., 2016; Shimpi et al., 2020). The Center for Disease Control and Prevention's National Prevention Program estimates that such interventions can lower the risk of developing T2DM by nearly 60% (70% after age 60 years) (Centers for Disease Control and Prevention, 2018). However, traditional silo‐ed medical and dental healthcare delivery models in the United States pose substantial barriers to holistic care delivery for the management of cumulative health risks associated with these conditions.

Following the review of 14 studies that collectively enrolled >32,000 patients, a 2018 consensus report by the International Diabetes Federation and European Federation of Periodontology concluded that a solid evidence base supported links between periodontitis and T2DM and its complications (Sanz et al., 2018). Moreover, a recent systematic review of 10 field trials exploring chairside screening of undiagnosed T2DM/prediabetes in the dental setting consistently demonstrated high levels of prediabetes across diverse dental primary care practices (Glurich, Bartkowiak, et al., 2018). These findings collectively promote the adoption of an alternative integrated health care delivery paradigm with focus on early detection and management of undiagnosed T2DM/prediabetes. Such an integrated care model would include cross‐disciplinary screening in both medical and dental settings with appropriate triage across the medical and dental domains.

Assessing patient risk for a diabetes involves noninvasive screening of patients by assessing their status with respect to informative candidate variables. Patient status for defined risk variables is assessed by risk prediction modeling applying statistical approaches including multiple regression modeling. Analysis of data available in medical electronic health records (EHR) that predict relative risk for diabetes include family history, clinical, pharmaceutical, demographic, and environmental factors. Data modeling holds promise for noninvasive detection of undiagnosed disease based on information already available in the EHR.

Historically, over 80 publications have reported on the evaluation of the performance of diabetes risk prediction models in various populations. However, few models have included oral health variables. Over the past 10 years as the evidence base suggesting a bidirectional association between T2DM and periodontitis progression increased, researchers began exploring the application of diabetes risk modeling in dental settings as a noninvasive approach to risk assessment. Such studies also tested risk prediction model performance incorporating dental variables and compared performance of these models to biological determination of glycemic levels as the gold standard (Borrell et al., 2007; Holm et al., 2016; Lalla et al., 2011, 2013; Li et al., 2011; Strauss et al., 2010).

A systematic review conducted by Collins et al. (2011) evaluated methodology used in studies that developed multivariable diabetes risk prediction models from 1980 to 2011, and included studies that had evaluated oral health factors. While search terms used by Collins et al. (2011) and our study varied somewhat, both search strategies identified the same articles within the temporal window of their study that had applied multivariable analysis. The current study included those studies also reviewed by Collins et al. (2011) that had applied rigorous modeling approaches and also met the eligibility criteria of the current study. However, the temporal frame of the current study was extended to include eligible studies published through July 31, 2018. The rationale for inclusion of later studies was to identify additional models that met inclusion criteria of the current study with the same stringency criteria applied by Collins et al. (2011) and further explored inclusion of oral health variables in predictive models examining risk for undiagnosed Type II diabetes/prediabetes. The systematic review was conducted on eligible studies that applied multivariable regression modeling or bioinformatics approaches with a requirement for inclusion of oral health/dental variables.

## 2 MATERIALS AND METHODS

### 2.1 Systematic review

The current study met exemption criteria of the Institutional Review Board in those research activities were limited only to the review of historically published literature and included no research activities involving human subjects or animals. The systematic review was conducted and reported in accordance with “preferred reporting items for systematic reviews and meta‐analyses” (PRISMA) statement (Moher et al., 2009). The research question in the current study was defined within the patients—(intervention/exposure/*prognostic factor*)—comparison group‐outcome) (PICO) framework as follows:

“P” = persons at risk for undiagnosed T2DM or prediabetes;

“I” = *prognostic factor*: oral health variable(s) alone or in combination with other risk variables;

“C” = *comparison group*: comparisons with a nondiabetic population; and

“O” = *outcome*: predictive capacity defined by the derived predictive model.

### 2.2 Literature identification selection and review

**Figure 1:**
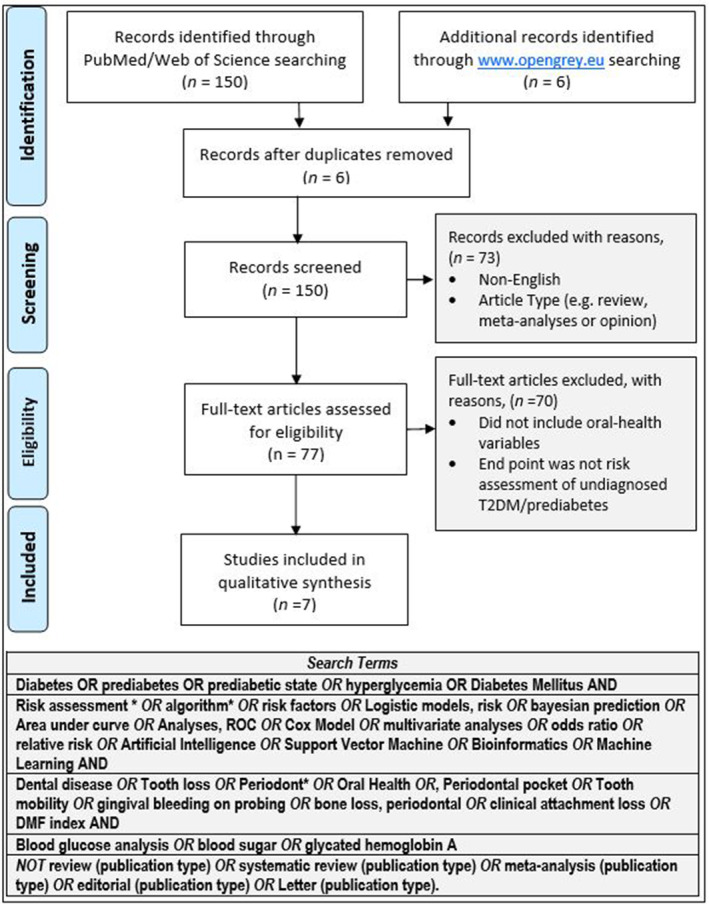
Search terms and PRISMA flow diagram. This figure summarizes the search terms used to identify potentially eligible publications and the outcome of the PRISMA review process denoting numbers of publications initially identified and screened for eligibility and the final number of publications meeting criteria for full systematic review. PRISMA, preferred reporting items for systematic reviews and meta‐analyses (Moher et al., 2009)

Articles published from January 1, 1980 through July 31, 2018 including those meeting statistical rigor previously defined in the systematic review by Collins et al. (2011) were identified by systematic searches conducted in PubMed, Web of Science databases, and Gray Literature with last search conducted in August, 2018. Literature was retrieved using the search strategy defined in Figure 1. Literature searches were conducted by a medical librarian with extensive expertise and experience in the conduct of systematic reviews.

Articles qualifying for inclusion were required to meet the following eligibility criteria.

#### 2.2.1 Inclusion criteria

Original articles were required to:

1. specify the creation of T2DM/prediabetes risk prediction models in undiagnosed individuals by modeling oral/dental and other candidate risk variables applying multivariable regression modeling or biomedical informatics approaches such as machine learning or classification and regression trees (CART) analysis;
2. include oral/dental health‐related variables;
3. be published between January 1, 1980 and July 31, 2018; and
4. report on model performance relative to predicting risk for T2DM/prediabetes. Articles that compared the performance of models that included oral and other candidate risk factors with and without biological screening outcomes were also retained and systematically reviewed.

#### 2.2.2 Exclusion criteria

Articles were excluded if they were a publication type other than an original article, predicted risk for other diabetes types, fell outside of the defined date range, were in a language other than English, did not include at least one oral health‐related variable, and did not evaluate predictive capacity of the models.

### 2.3 Template development, data collection, inter‐rater reliability, and quality assessment

**Figure 2:**
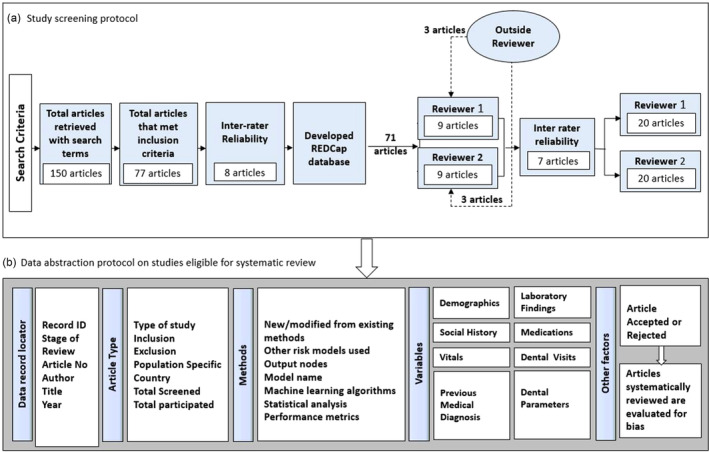
Flow chart of data abstraction and systematic review protocol. (a) Provides an overview of the study screening protocol including the determination of inter‐rater reliability. (b) Summarizes the abstraction protocol including publication types screened, screening of methodological approach and variables assessed by the publications, and conduct of the systematic review and bias evaluation applying PRISMA and PROBAST on articles meeting eligibility criteria. PRISMA, preferred reporting items for systematic reviews and meta‐analyses; PROBAST, prediction model risk of assessment bias tool

A review protocol (Figure 2) was created to ensure systematic abstraction and data capture across each manuscript. The template was pilot tested by three reviewers to ensure cohesive understanding of the definition of all abstraction terminology among reviewers. Title and abstract review were first conducted to identify articles requiring further review. Following the initial review of *n* = 8 articles by two reviewers (N.S. and I.G.), inter‐rater assessment as done to evaluate the interpretation of defined abstraction terms and inform finalization of the research electronic data capture (REDCap) (Harris et al., 2009) template used for full‐text review of remaining articles. A second inter‐rater assessment of an additional three articles tested agreement between data collected via the REDCap‐based template used by each reviewer. Quality assessment was further conducted following abstraction of the same articles by a third reviewer (G.J.) to replicate good inter‐rater agreement using the template. The final inter‐rater assessment was conducted to confirm high inter‐rater agreement (*n* = 7) between the reviewers (N.S. and I.G.), and was reported as the percentage agreement between reviewers. Summary measures assessed included outcomes of predictive modeling. The most frequent analyses presented included area under the curve (AUC), and/or assessment of sensitivity, specificity, and positive and negative predictive values.

### 2.4 Bias assessment

The risk of bias was assessed by two reviewers (I.G. and R.B.). The prediction model risk of assessment bias tool (PROBAST) (Wolff et al., 2019) was applied. The PROBAST tool was published in 2019 specifically to address the absence of a bias assessment tool for studies developing or validating predictive models.

## 3 OUTCOMES OF SYSTEMATIC REVIEW

Search terms and outcomes of the systematic search strategy summarized in the PRISMA flow chart are shown in Figure 1. Among 141 articles retrieved, 95 articles defined candidate variables for predicting risk for undiagnosed T2DM/prediabetes, and 71 studies met eligibility for full review. Among the 71 articles, 10 studies were identified that included oral health or dental variables. However, three were found to be review articles or commentaries and were excluded. Thus, seven publications met the criteria and were systematic reviewed. Inter‐rater agreement across data elements abstracted during final assessment achieved 99%.

**Figure 3:**
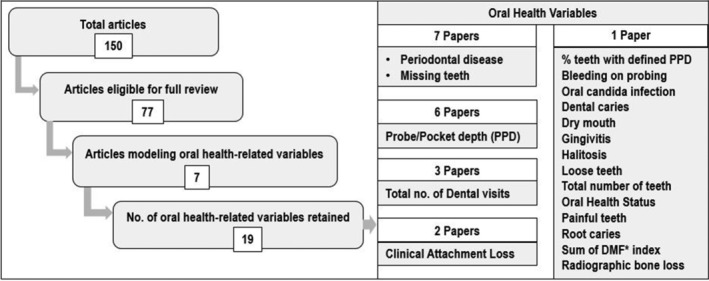
Oral health‐related variables identified by the systematic review. This figure summarizes the 19 oral health variables that were evaluated among articles systematically reviewed. Two variables consistently identified and retained in models by all seven articles systematically reviewed included the number of missing teeth and presence of periodontitis based on documentation of variables associated with pathophysiological manifestations of periodontitis

Table 1 summarizes an overview of the outcomes of the systematic review. Collectively, the studies demonstrated that oral variables contribute to models that predict the presence of undiagnosed T2DM/prediabetes identified by biological testing, both independently and in combination with other candidate risk factors. At the conclusion of systematic review, it was determined that further meta‐analysis was not feasible based on detection of high variability across study design of the seven reviewed studies, goal and outcomes, study population characteristics, and settings in which these studies were performed. Oral/Dental variables (*n* = 19) modeled in the systematically reviewed publications, and the number of publications that modeled each variable are cataloged in Figure 3. Variables most consistently retained in the final models included the number of missing teeth, periodontal probing depth (PPD), and/or clinical attachment loss (CAL) measures that indicated the presence of severe periodontitis.

**Table 1:** Undiagnosed T2DM/prediabetes prediction models incorporating both clinical and oral health‐related variables

Author	Study Objective	Periodontitis definition	Type,# models	Variables retained, other measures	Performance metrics	Other notes
Borrell^19^(2007)	Modeled conditional undiagnosed T2DM/prediabetes prediction (defined as FPG>126mg/dL) using NHANES III data for subjects >20 yrs. of age and compare performance across race/ ethnicity in patients with no DM dx (n=4830 analyzed)	2 definitions were tested: 1) > 2 sites w/ CAL; > 6mm and >1 site with PPD >5 mm at a max of 14 teeth at 28 sites screened in two quadrants at 2 sites/tooth (mid & mesiobuccal) 2) > 2 sites with PPD> 5mm or 1 site with PPD > 4,5,6, or 7mm	LRA used to estimate conditional probability of undiagnosed DM. Variables analyzed: age, sex, ethnicity/race, family hx of DM (parents/ sibs); htn (self‐report), periodontitis, hypercholesterolemia. Two models were created using two distinct periodontitis definitions.	Age>45yrs; sex, ethnicity/race, family hx of DM (parents/sibs); HTN (self‐report), periodontitis, hypercholesterolemia. Inter‐group comparisons based on race/ethnicity used *X* ^*2*^ or t test.	Sensitivity: mean AUC=0.76; In African Americans: AUC=0.81; In Mexican‐Americans: AUC=0.76 Models had 27‐53% probability of predicting DM	Analyses were adjusted for # of teeth
Holm^24^ (2016)	Identify previously undiagnosed patients with probable DM or pre DM in dental setting with POC screening for HbA1c>39‐47 mmol/mol for prediabetes or > 48mmol/mol for T2DM; and referral for diagnostic testing to confirm status (n=291)	Based on full‐mouth exam and radiographic bone loss measure. Periodontitis definition: >2 interproximal sites with CAL >6mm, and >1 interproximal site with PPD>5mm. Other dental variables tested: BOP, RBL DMFT: (p = 0.000) for each of these dental variables	LRA to predict DM: 3 models: HbA1C measure in combination with: Model 1: One interproximal site PPD > 5mm +BMI (overweight, obese), Model 2: Two interproximal sites AL > 6mm + 1 site with PPD >5mm + BMI (overweight, obese); Model 3: 1 site with PPD >5mm + waist circumference (high risk)	Variables retained: Periodontitis, (defined by >1 site PPD > 5mm or 2 sites > 6mm not on same tooth); BMI (obese or overweight); WC; fat % Intergroup comparisons between patients with or without periodontitis, used *X* ^*2*^ or Fischer exact test.	**Table:** Sens Spec PPV NPV PD 0.91 0.19 0.33 0.83 Fat % 0.74 0.55 0.41 0.83 WC 0.60 0.68 0.45 0.80 PD+BMI 0.42 0.81 0.68 0.60 PPD+BMI+ fat%+WC 0.48 0.78 0.49 0.78 Model 1 AUC 0.656 (CI:0.59‐0.72) Model 2 AUC 0.651 (CI:0.58‐0.71) Model 3 AUC 0.657 (CI:0.59‐0.72)	Overweight/obese (BMI) and overweight/obese fat % and WC also showed predictive capacity for detection of periodontitis.
Lalla^20^ (2013)	Define rates of undiagnosed T2DM/prediabetes by: 1)POC screening for HbA1C> 6.5% in dental setting with definition of periodontitis status 2) evaluate performance of 3 predictive models for undiagnosed T2DM/prediabetes for patients with available POC glycemic level data and oral status data (n=591 subjects analyzed).	>4 missing teeth and 26% of teeth with PPD >5mm	ROC evaluation of predictive models for undiagnosed T2DM/prediabetes through application of logistic regression analysis Three predictive models were applied after assessing for optimal cutoff points: 1) Model 1: predictive capacity of oral criteria ( >26% teeth with at least one deep pocket or >4 missing teeth); 2) Model 1 plus POC HbA1C >5.7; 3) POC HbA1C alone	Variables assessed: (Hispanic subject: age >30 years; white subject: Age >40 yrs); plus 1 additional risk factor (family hx of DM, HTN, dyslipidemia, or BMI>25kg/m2), Periodontitis, (defined by PPD> 5mm), > 4 missing teeth. Inter‐group comparisons between normo‐ and hyper‐glycemic used *X* ^*2*^ or Fischer exact test.	1) predictive potential surrounding percent of teeth with at least one PPD > 5mm and > 4 missing teeth + risk factors (Family hx of DM, htn, dyslipidemia, BMI (overweight/obese) (AUC= 0.58) 2) model 1 plus POC HbA1C >5.7% (AUC=0.92) 3) POC HbA1C > 5.7% alone (AUC=0.92) **Table:** Model Sens Spec PPV NPV 1 0.72 0.37 0.58 0.52 2 0.87 0.35 0.62 0.69 3 0.62 0.96 0.95 0.67	Performance of models #1&2 on combined data for Lalla^20.21^ for 1097 subjects: #1) predictive potential surrounding percent of teeth with at least one PPD>5 measure and > 4 missing teeth (AUC=0.60; CI 0.56‐0.63) #2) model 1 plus undiagnosed T2DM/prediabetes defined by FPG or HbA1c (AUC=0.83; CI 0.80‐0.85) (p<0.0001) **Table:** Sens Spec PPV NPV 1 0.72 0.37 0.58 0.52 2 0.87 0.35 0.62 0.69 3 0.62 0.96 0.95 0.67
Lalla^21^(2011)	Define rates of undiagnosed T2DM/prediabetes by: 1)POC HBA1C screening in dental setting with definition of periodontitis status and confirmation by FPG > 100mg/dL 2) evaluate performance of 3 predictive models for undiagnosed T2DM/prediabetes for patients with available POC glycemic level data and oral status data (n=506 subjects analyzed).	> 4 missing teeth and 26% of teeth with PPD >5mm, BOP	ROC evaluation of predictive models for undiagnosed T2DM/prediabetes through application of LRA* Three predictive models were applied after assessing for optimal cutoff points: 1) Age: (Hispanic subject: >30 years; white subject: >40 yrs) + % PPD >5mm +BOP+Fam hx of DM +HTN +dyslipidemia +BMI (overweight/obese) (AUC 0.68) 2) % PPD> 5mm + # of missing teeth (AUC 0.65) 3) Model 2 parameters + POC HbA1C (AUC 0.79)	Hispanic subject: age >30 years; or white subject: Age >40 yrs); plus 1 additional risk factor (family hx of DM, HTN, dyslipidemia, or BMI>25kg/m2. Periodontitis (defined by PPD >5mm), > 4 missing teeth. Inter‐group comparisons between normo‐ and hyper‐glycemic used *X* ^*2*^ or Fischer exact test.	1) predictive potential surrounding percent of teeth with at least one PPD>5mm and >4 missing teeth + risk factors (Family hx of DM, htn, dyslipidemia, BMI (overweight/obese) (AUC= 0.68) 2) model 1 plus POC HbA1C >5.7% (AUC=0.65) 3) POC HbA1C > 5.7% alone (AUC=0.92) **Table:** Model Sens Spec PPV NPV 1 0.73 0.45 0.43 0.75 2 0.92 0.28 0.42 0.86 3 0.75 0.56 0.49 0.80	Data from 2011 study were combined and remodeled with data from Lalla (2013); population modeled had high % of Hispanic ethnicity
Herman^32^ (2015)	Derive a screening model for detecting undiagnosed T2DM/prediabetes at POC screening for HbA1c > 5.7% in the dental setting in patients with no previous dx; conduct risk factor analysis and test referral for diagnostic validation (n=1033)	Criteria: pain, periodontitis, dx mobility, >1 missing tooth, or applying clinical classification scoring using a scale of 1 to 5 where a score of 3=mild, 4=moderate, 5=severe)	LRA testing potentially predictive variables including: age, sex, race/ethnicity, education, income, self‐ reported: height, weight, physical activity; hx of: CVD, dyslipidemia or treatment, htn or treatment, smoking, gestational diabetes; family hx of DM, insurance coverage, access to medical care, self‐reported periodontitis, symptomology; hx of tooth loss, capillary glucose measure >110mg/dL. Subset of patients with capillary glucose >110mg/dL or assessment of periodontitis, (defined as: mild moderate, severe) also had HbA1C test. HbA1C interpretation: >5.7‐6.4= prediabetes; HbA1C >6.4=DM	Apply age and BMI as continuous variables, final model included: sex, HTN, dyslipidemia, > 1 missing tooth, capillary glucose >110mg/dL. Significant association noted between HTN & sex. Model explained 24% undiagnosed T2DM/prediabetes likelihood *X* ^*2*^. Factors independently associated with pre‐ DM or putative DM following removal of capillary glucose from the model included: BMI > 35kg/m2 (OR=2.6; p=0.064); males with htn (OR=1.4, p=0.32); females with HTN (OR 4.5, p=0.11) dyslipidemia (OR=5.3; p<0.001,) > 1 missing tooth (OR=2.5, p=0.012), BMI >35kg/m2 (OR=2.6, p=0.064). Model explained 20% of undiagnosed DM/pre‐DM likelihood *X* ^*2*^	A cut point of specificity=80% was set to reduce number of false positives. The undiagnosed T2DM/prediabetes predictive models included: Model 1: risk factors with inclusion of cap gluc >110mg/dL: AUC=0.83 (sensitivity=60%); Model 2: with risk factor and without inclusion of cap gluc >110mg/dL: AUC =0.79 (sensitivity=50%)	A cut point of ‐0.087 was calculated from the logistic regression model (corresponding to a probability of 0.45), to achieve a specificity of 80% (sensitivity=50%)
Li^23^(2011)	Propose clinical practice guideline to help assess undiagnosed dental patient risk for DM following screening for FPG > 126mg/dL by modeling predictive variables of subjects in NHANES III (n=15,090)	NHANES III definitions [25]: assessment of two or more sites with CAL > 3mm, and >1 site with PPD=4mm, gingival recession assessing two quadrants (maxillary and mandibular at two sites per tooth (buccal and mesio‐buccal)	55 potentially predictive variables were evaluated by applying CART non‐parametric statistical analysis to 50% of data set to develop the diabetes model and conduct of internal validation in the other 50% of data	Retained predictive variables of undiagnosed T2DM/prediabetes: periodontitis, oral health status, DMFT, race/ethnicity, age, poverty income ratio, educational level, time since last medical visit, time since last dental visit, physical activity, BMI, weight, height, WC (self‐ reported), CHF, HTN, serum cholesterol, triglycerides HDL, CRP.	**Table:** Training 10X X‐valid Int Valid Ext Valid sens 91.2 82.4 79.8 82.4 spec 52.2 51.8 52.1 52.8 AUC 0.74 0.68 0.70 0.72	Developed a flow chart to determine relative risk and if patient should be screened or triaged using the following pivotal variables: 1) waist circumference (>35 inches (90cm) (self‐reported); 2) age >45 years (or >36yrs for Hispanic ethnicity) 3) self‐reported weight (> (68kg)) 4) self‐reported black race or Hispanic ethnicity 5) poor oral health
Liljestrand^25^ (2015)	Evaluate # of missing teeth as a predictive variable for incident DM & all‐cause mortality in a cohort (n=7198) with 13 years of follow‐ up. DM dx was based on questionnaire reporting MD‐diagnosed DM and querying the disease‐associated drug reimbursement records for drug use associated with glycemic management from the Social Insurance Institution of Finland	number of missing teeth	Cox regression modeling to evaluate significance (calculated by Mann‐Whitney U or *X* ^*2*^ ) surrounding selected variables and disease incidence over a 13‐year temporal frame evaluated by # of missing teeth: Variables adjusted for included: age, sex, smoking, no DM at baseline; age; sex; BMI; sedentary lifestyle; family hx of DM (parents); log CRP. Analysis of linear trend in a one‐way analysis of variance was also performed.	Adjusted covariates retained in compliance with DM risk score [27] ): age (>45 years), waist circumference (♀>87cm, ♂>101cm) BMI (overweight, obese), htn med hx; hx of elevated blood glucose; sedentary life style (<4hrs/week of activity); daily fruit and vegetable consumption. Model discriminatory capacity was tested by including/excluding missing teeth as a variable. Significant linear relationship with # of missing teeth was associated with: age, education (inverse to # of years), BMI, CRP, HDL cholesterol, triglycerides, male sex, existing DM, parental hx of DM or MI.	Looked at HR for mortality based on # of missing teeth: Between 9 to31 missing teeth: HR=1.37 (p=0.040) Edentulous: HR=1.56 (p=0.012)	Increased risk for DM was observed beginning at >5 missing teeth

Abbreviations: LRA: Logistic regression analysis; hx: history of; yrs: years; POC: point of care, DM: diabetes mellitus, MI: myocardial infarction, CVD: cardiovascular disease, HTN: hypertension BP: blood pressure, sBP: systolic BP, dBP diastolic BP; PD: periodontitis; PPD: periodontal probing depth; CAL: clinical attachment loss, RBL radiographic bone loss; BOP:bleeding on probing, DMFT: decayed, missing, filled teeth, BMI: body mass index; Chol(tot)=total cholesterol; HDL: high density lipoprotein, LDL: low density lipoprotein; CRP: C‐reactive protein, HR: hazard ratio; AUC: area under the curve; ROC: receiver operator characteristic curve; yr(s)=year(s); #=number; CHF=congestive heart failure; FPG=fasting plasma glucose; WC=waist circumference; fat%=fat percentage; sens=sensitivity; spec=specificity; PPV=positive predictive value; NPV=negative predictive value; dx=diagnosis; 10X‐X‐valid=ten‐fold cross validation; Int Valid=internal validity; Ext Valid=external validity; HR=hazard ratio; MI=myocardial infarction; RX=prescription medication

Results of bias assessment applying the PROBAST are shown in Table 2. Potential sources of bias associated with most studies included the use of self‐reported data and incomplete reporting of analytical detail. The risk of bias or applicability consistently ranged from “low” to “unclear” across studies systematically reviewed.

**Table 2:** PROBAST bias assessment results table

Study	ROB				APP				Overall		Observations
	Participants	Predictors	Outcome	Analysis	Participants	Predictors	Outcome	Analysis	ROB	APP	
Borrell et al. (2007)	+	?	+	?	+	?	+	?	?	?	Discovery only; Low event rate/group; Low predictive power to detect the probability of T2DM (27%–53%); Algorithm was published
Li et al. (2011)	+	?	+	?	+	?	+	?	?	?	Discovery‐used NHANES III cohort data CPG development was goal; model was internally and externally validated; rates of Type I/Type II error not reported. Set predictive cutoff was arbitrary; clinical utility unclear
Lalla et al. (2011)	+	?	+	+	+	?	+	?	+	+	Discovery only; Modeling assumptions and criteria not specified; predictor weights and regression coefficient were not reported; model can discriminate across three outcomes; holds probable clinical utility
Lalla et al. (2013)	+	?	+	+	+	?	+	+	+	+	Validation; combined new data with data from Lalla et al. (2011) study. High AUC; supports clinical utility; model not published; no validation of DM diagnosis reported.
Herman et al. (2015)	+	+	+	+	?	?	+	+	?	?	Discovery only; model predicted risk for DM by 10 years age groups and sex; Increased AUC of biological measure alone; specificity was 80%, sensitivity 60%; low response to follow‐up biological testing to validate DM; model not validated or published
Liljestrand et al. (2015)	+	+	+	+	+	+	+	+	+	?	Discovery only; Modeled candidate predictors with longitudinal data; demonstrated # of missing teeth as predictor of DM in Finnish population; No model was published
Holm et al. (2016)	?	+	+	?	?	+	+	?	?	?	Discovery only; Self‐reported data not modeled; modeling assumptions and testing/outcomes were not reported; Biological measure was not modeled to assess the impact of performance

*Note*: Scoring code.

Abbreviations: APP, applicability; AUC, area under the curve; CPG, clinical practice guideline; PROBAST, prediction model risk of bias assessment tool (Wolff et al., 2019); ROB, risk of bias; +, low risk of bias or probable applicability; ‐, high risk of bias or low applicability; ?, unclear risk of bias and uncertain applicability.

## 4 DISCUSSION

The current study undertook a systematic review of the application of predictive modeling for detection of undiagnosed T2DM/prediabetes applying oral health variables as candidate predictors. This study builds upon an earlier study by Collins et al. (2011), which had systematically examined the methodological rigor of studies published through 2010 whose goal was the prediction of undiagnosed T2DM/prediabetes. Guided by the outcomes of their analysis, only studies that applied multivariable regression modeling to define predictive variables were considered for inclusion.

Most multivariable regression modeling across studies systematically reviewed was generally undertaken as “proof of concept” to test predictive model performance as a surrogate for biological testing in the clinical setting where such models may have applicability for screening patients to detect risk for undiagnosed disease. Studies reported mainly on model development and performance in the population under study and cited need for further validation and testing of their models across other populations to assess clinical applicability and portability to other settings.

Whereas all of the studies systematically reviewed herein focused on assessing the prediction of undiagnosed T2DM/prediabetes using dental variables alone or in combination with other predictive variables, the objectives of the studies varied. Studies by Holm et al. (2016) and Lalla et al. (2011, 2013) explored point of care (POC) glycemic screening in the dental setting to define rates of undiagnosed T2DM/prediabetes, while Borrell et al. (2007) and Herman et al. (2015) modeled data to project population‐based levels of undiagnosed T2DM/prediabetes across time. Li et al. (2011), undertook predictive modeling with the purpose of developing clinical practice guidelines and was the only study to apply CART analysis. Li et al. (2011) and Borrell et al. (2007), both applied predictive modeling approaches to NHANES data. Due to high variability in the study populations, outcomes, and study design, formal meta‐analysis of the studies was precluded. Variability across the studies was noted with respect to: (1) population characteristics, (2) timing on follow‐up for glycemic testing in the medical setting; and (3) use of data from partial mouth assessment versus full‐mouth assessment of periodontitis. Eke et al. (2010), previously reported that partial mouth assessment may underestimate the true prevalence of periodontitis. Further, bias assessment with the recently published PROBAST (Wolff et al., 2019) tool further found “low” (*n* = 3) to “unclear” (*n* = 4) risk of bias and probable (*n* = 1) to uncertain (*n* = 6) applicability across studies, since 6/7 studies presented only outcomes of modeling without publishing the final models and only 1/7 models was further validated.

Notably, periodontitis‐associated measures modeled as risk factors were consistently retained in the predictive models, thus further reinforcing the evidence base supporting bidirectional interaction between undiagnosed T2DM/prediabetes and the infectious/inflammatory processes associated with periodontitis. Two variables: “PPD” and/or “number of missing teeth” were retained in all seven models as predictors in all populations in which they were tested. Apart from trauma, or tooth loss associated with extractions associated with either oral oncology‐related treatment or caries, periodontitis represents the most likely cause of missing teeth and is, therefore, a surrogate for prior history of periodontitis. Periodontitis‐associated tooth loss is attributable to loss of structural support due to damage of surrounding gums and underlying bone caused by chronic periodontal infection and host immune response to the local infectious processes in the gingival tissue. Consistent retention of these two related periodontal variables suggests high generalizability since they were identified by all seven articles across highly variable study populations and dental care settings.

Further, studies that modeled oral variables independently as the main predictors of risk for undiagnosed T2DM/prediabetes found these variables to be highly associated with biological outcomes. Analyses of combined data sets from two studies by Lalla et al. (2011, 2013), reported high sensitivity (0.87) and area under the receiver operating curve (AUC) (0.83) with use of an algorithm including only two optimally defined oral variables: ≥26% of teeth with deep pockets (defined as ≥5 mm and ≥four missing teeth) and glycemic measure (HbA1C) collected at the POC.

As shown in Table 1, the performance of models incorporating oral health‐related variables achieved AUC measures of up to 0.92. Model performance was further enhanced following the inclusion of biological glycemic screening measures as predictive variables during modeling. These results suggest that such predictive models merit further investigation and evaluation as potential tools for diabetic risk prediction in the dental setting.

Because predictive modeling holds potential for the creation of noninvasive tools for identifying individuals at risk for diabetes/prediabetes, which has achieved epidemic status, future studies should aim to follow a standardized study design to support testing of portability of predictive models with inherent potential for translation into clinical practice settings. Notably, among all studies systematically reviewed, only Borrell et al. (2007) published their final algorithm, thereby limiting further validation of these models in other populations. Among studies that also propose the inclusion of biological glycemic measures in predictive modeling standardization of screening protocols at POC is further required as identified by a previous systematic review (Glurich, Bartkowiak, et al., 2018). Thus, confirmation of accuracy of positive and negative predictive value of screening measures remains to be validated by longitudinal glycemic assessment.

Predictive modeling approaches reviewed herein identified variables that potentially contribute to risk for detecting the presence of undiagnosed disease. Monitoring of these variables at POC may have clinical applicability particularly in the dental setting. These findings further emphasize the value of monitoring oral health in both the dental and medical setting with appropriate triage for cross‐disciplinary care. Applicability of informatics to create clinical decision support tools with capacity for monitoring predictive clinical and demographic variables standardly collected in the EHR in the course of care delivery may be leveraged to facilitate identification of undiagnosed individuals requiring follow up and clinical management. Implementation of such tools in clinical and dental settings has recently been reported (Acharya et al., 2018; Hegde et al., 2019). While risk prediction modeling is data‐driven and noninvasive, studies by Lalla et al. (2011, 2013) further demonstrated enhanced predictive power (increase in AUC) when oral predictive variables were modeled in conjunction with biological measures of glycemic status as compared to models that did not use oral health variables.

### 4.1 Limitations

Limitations of the current study included the inability to conduct meta‐analysis based on high degree of variability across study populations, outcomes, and study designs. Further, the ability to conduct literature searches in other databases and languages may have resulted in lack of capture and inclusion of all articles meeting inclusion criteria for this review.

## 5 CONCLUSION

This systematic review defined oral health variables with attributable risk for undiagnosed T2DM/prediabetes. Notably, some studies demonstrated that oral health variables contributed to models predicting risk undiagnosed T2DM and prediabetes both independently and in combination with biological glycemic measures. The most consistently informative variables across studies included number of missing teeth and demonstration of periodontitis, defined by PPD and CAL. Findings of the current systematic review reflect those of a recently published systematic review of field trials that undertook screening for undiagnosed T2DM/prediabetes at POC in dental settings (Glurich, Bartkowiak, et al., 2018) that collectively reported high prevalence of undiagnosed T2DM/prediabetes across various populations applying POC testing alone or in combination with predictive modeling.

The outcomes of this systematic review supports that the application of noninvasive informatics approaches that model available data in the EHR and/or other administrative databases inclusive of oral health‐related variables could have value in identifying individuals with undiagnosed diabetes/prediabetes in the dental setting. Whereas all studies demonstrated the ability to model risk status, studies varied broadly in their design and purpose. Future studies using informative variables identified by these studies applied to different populations utilizing a standardized study design to support future meta‐analysis would be required to determine whether broadly applicable algorithms with high portability could be defined for more generalizable translation to clinical settings where determination of validity to the population has been demonstrated. Once validated, such models could be incorporated into EHRs in the form of clinical decision support tools if data on the required variables included in the models is available in the captured EHR data to support the performance of screening and predictive modeling.

## CONFLICT OF INTEREST

The authors declare no potential conflict of interest.

## Data Availability

Data sharing not applicable ‐ no new data generated.
